# RNA sequencing suggests that non‐coding RNAs play a role in the development of acquired haemophilia

**DOI:** 10.1111/jcmm.17741

**Published:** 2023-06-14

**Authors:** Adrian Bogdan Tigu, Ionut Hotea, Rares Drula, Alina‐Andreea Zimta, Noemi Dirzu, Maria Santa, Catalin Constantinescu, Delia Dima, Jon Thor Bergthorsson, Victor Greiff, Diana Gulei, Daniel Coriu, Margit Serban, Johnny Mahlangu, Ciprian Tomuleasa

**Affiliations:** ^1^ Medfuture Research Center for Advanced Medicine Iuliu Hatieganu University of Medicine and Pharmacy Cluj Napoca Romania; ^2^ Department of Hematology Iuliu Hatieganu University of Medicine and Pharmacy Cluj Napoca Romania; ^3^ Department of Hematology Ion Chiricuta Clinical Cancer Center Cluj Napoca Romania; ^4^ Biomedical Center, School of Health Sciences University of Iceland Reykjavík Iceland; ^5^ Department of Immunology University of Oslo and Oslo University Hospital Oslo Norway; ^6^ Department of Hematology Carol Davila University of Medicine and Pharmacy Cluj Napoca Romania; ^7^ Department of Pediatrics Victor Babes University of Medicine and Pharmacy Timisoara Romania; ^8^ Haemophilia Comprehensive Care Centre Charlotte Maxeke Johannesburg Academic Hospital, University of the Witwatersrand and National Health Laboratory Service Johannesburg South Africa; ^9^ Romanian Academy of Scientists Bucharest Romania

**Keywords:** acquired haemophilia, coagulation, epigenetics, factor VIII, RNA sequencing

## Abstract

Acquired haemophilia (AH) is a rare disorder characterized by bleeding in patients with no personal or family history of coagulation/clotting‐related diseases. This disease occurs when the immune system, by mistake, generates autoantibodies that target FVIII, causing bleeding. Small RNAs from plasma collected from AH patients (*n* = 2), mild classical haemophilia (*n* = 3), severe classical haemophilia (*n* = 3) and healthy donors (*n* = 2), for sequencing by Illumina, NextSeq500. Based on bioinformatic analysis, AH patients were compared to all experimental groups and a significant number of altered transcripts were identified with one transcript being modified compared to all groups at fold change level. The Venn diagram shows that haemoglobin subunit alpha 1 was highlighted to be the common upregulated transcript in AH compared to classical haemophilia and healthy patients. Non‐coding RNAs might play a role in AH pathogenesis; however, due to the rarity of HA, the current study needs to be translated on a larger number of AH samples and classical haemophilia samples to generate more solid data that can confirm our findings.

## 1 INTRODUCTION

Acquired haemophilia (AH) is a very rare disease, in which autoantibodies produced in an associated underlying condition inactivate FVIII.^1^ Cutaneous purpura and internal bleeding are the main clinical manifestations.^2^ Diagnosis is based on measuring FVIII concentration in plasma, in the presence of inhibitors, while the treatment is mostly focused on inhibiting the bleeding episodes.^3^, ^4^

In AH, epigenetics could play an important role in both deciphering non‐mutational and mutational status of *F8* gene, as well as how autoimmunity is triggered against FVIII. As noncoding molecules can interact with *F8* gene and non‐genetic events may cause AH, non‐coding RNAs potentially influence *F8* expression and can trigger autoimmunity against FVIII. However, the impact of non‐coding RNAs in *F8* gene modulation is still unclear, due to the contradictory results previously reported.^5^, ^6^ The methylation pattern of *F8* gene and the surrounding regions could be attributed to HA severity. Yes, studies on family members with *F8* mutations showed that they express different degrees of severity, while other studies showed no difference between controls and haemophilic patients regarding their methylation pattern. Thus, due to the high variability in *F8* mutational status, the link between epigenetics and HA must be further investigated and certain interactions must be confirmed.^7^, ^8^, ^9^, ^10^

In our study, we investigated the transcriptomic signature in RNAs from the plasma of two AH patients, six classical haemophilia patients (three mild and three severe) and compared them to two healthy donors; by RNA‐sequencing to highlight relevant differentially expressed transcripts. We observed that haemoglobin subunit alpha 1 and both coding and non‐coding RNAs show different expressions in AH compared to all other investigated groups, potentially being a reliable biomarker candidate for predicting AH development. This might provide an insight into the altered biological pathways involved in AH development, which may be subject for further studies.

## 2 MATERIALS AND METHODS

### 2.1 Sample collection

As previously published protocols for serum and plasma isolation, the peripheral blood was collected on K_2_EDTA‐coated vacutainer and the plasma was immediately separated by and stored at −80°C.^11^, ^12^ Both AHA cases had no pre‐existent comorbidities and were diagnosed as idiopathic AHA, not related to any underlying condition.

### 2.2 RNA sequencing

Small RNA Seq libraries were generated using the ‘TrueQuant smallRNA‐Seq Kit’ for ultra‐low input material by GenXPro GmbH in Frankfurt am Main, Germany, according to the manual of the manufacturers. After silica column‐based isolation of small RNA from 500 μL of EDTA‐blood‐plasma, adapters including unique molecular Identifiers (patent number 102008025656) were ligated to both ends of the RNA. After cDNA generation and second‐ strand synthesis, PCR with minimum number of cycles was used to produce a library that was sequenced on an Illumina NextSeq500 machine with 1× 75 bps.

### 2.3 Bioinformatics workflow for small‐RNA seq

Raw data was pre‐processed using Cutadapt^13^ to eliminate low‐quality reads. FastQC was used to assess the quality of sequencing after trimming. Cleaned reads were mapped to the human reference genome GRCh38 using Bowtie2^14^ and to the miRbase (https://www.mirbase.org/). Quantification of mapped reads to each gene was performed using HT‐seq.^15^ Differential expression analysis was performed using DESeq2,^16^ which is based on negative binomial generalized linear models. Results were compiled into a final table including significance parameters (*p* value, FDR) and log2FoldChanges (‘all comparisons merged table’). Final data visualisation of the significantly expressed and the up/down‐regulated genes was performed by custom R‐Scripts.

### 2.4 Statistical analysis

The normalized values were compared using Welch's *t*‐test. A comparison was considered significant if the log2 FC was over one and the unadjusted *p*‐value was under 0.01.

## 3 RESULTS

**FIGURE 1:**
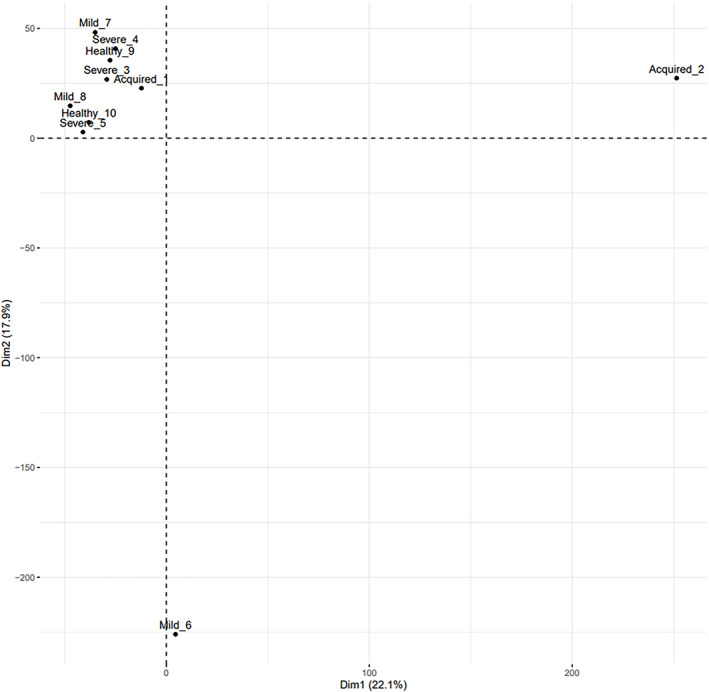
Principal component analysis of the included samples from AH patients (Aquired_1, Acquired_2), and mild classic haemophilia (Mild_6, Mild_7, Mild_8), severe classic haemophilia (Severe_3, Severe_4, Severe_5) and healthy donors (Healthy_9 and Healthy_10).

**FIGURE 2:**
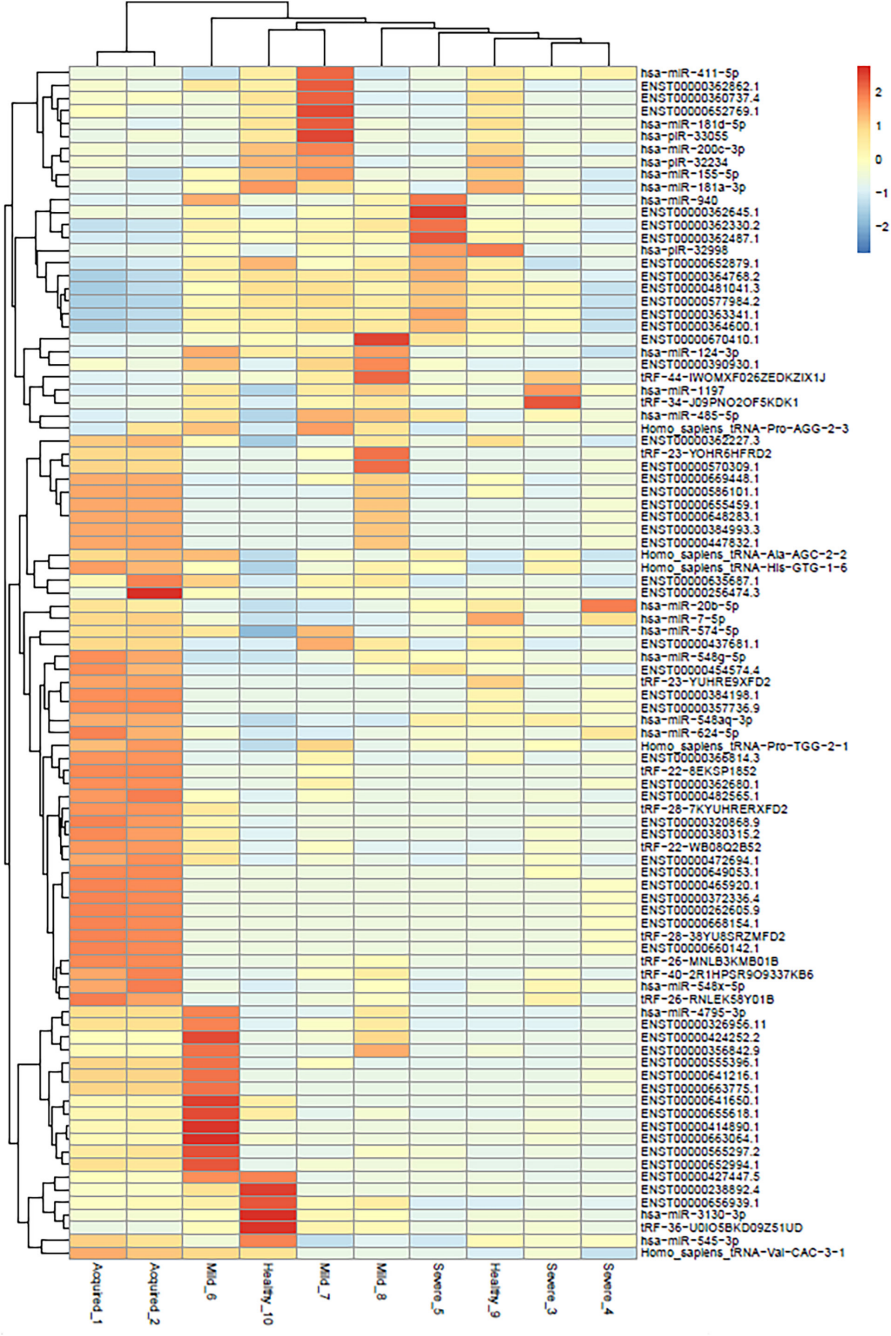
Heatmap of the small RNAs that were significantly different between any of the conditions. Comparison between samples from AH patients (Aquired_1, Acquired_2), and mild classic haemophilia (Mild_6, Mild_7, Mild_8), severe classic haemophilia (Severe_3, Severe_4, Severe_5) and healthy donors (Healthy_9 and Healthy_10).

**FIGURE 3:**
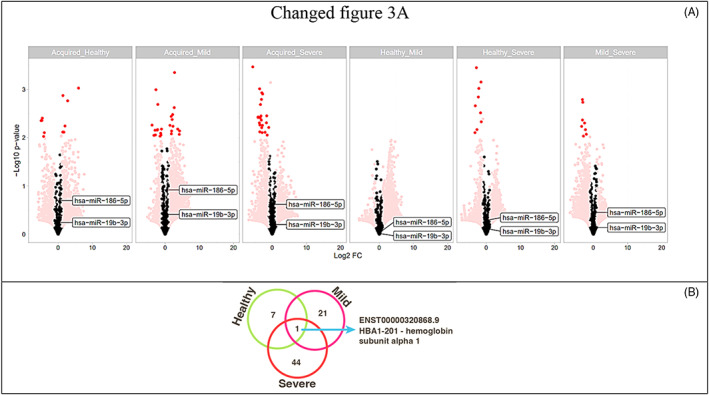
Volcano plots highlighting the global transcriptomic modifications and main altered transcripts (dark red) of all comparisons (A). Venn Diagram depicting the common upregulated transcripts in AH compared to each group. HBA1‐201 – haemoglobin subunit alpha 1 being the common transcript in AH compared to all groups (B).

The transcriptomic signature in the RNA isolated from plasma of AH patients (*n* = 2; Aquired_1, Acquired_2), and mild classic haemophilia (*n* = 3; Mild_6, Mild_7, Mild_8), severe classic haemophilia (*n* = 3; Severe_3, Severe_4, Severe_5) and healthy donors (*n* = 2; Healthy_9 and Healthy_10) (Figure 1) showed dysregulated transcripts were clustered in heatmaps which highlight modifications at fold change level (Figure 2) while the global comparisons of the alterations are displayed as volcano plots in Figure 3A.

A significant number of altered transcripts were observed when comparing AH to healthy donors and classical haemophilia patients. As presented in Figure 3A, the cutoff of the FC > 2 was used to select the most relevant differentially expressed transcripts. Based on the different transcript patterns it is visible that AH show different expressions than all other groups, which may lead us to a new path which can indicate potential transcripts that can act as future biomarkers.

After comparing all samples and determining which transcripts are altered between the groups, different subsets of transcripts (both coding and non‐coding) may act as start points for future investigations in the associated mechanisms. As represented in Figure 3B, haemoglobin subunit alpha 1 (HBA1‐201) is one of the specific upregulated transcripts in AH when compared to all other conditions. HBA1 may indicate a compensatory physiological response to acute blood loss.

HBA1 is located on chromosome 16 and has around 30 kb with two identical coding sequences alpha 2 and alpha 1, encoding the two alpha and betta chains of HbA. Previous data shows that deletions in HBA1 and HBA2 lead to deficit in haemoglobin and thalassemia.^17^, ^18^ Each non‐coding RNA can interact with multiple genes and one gene can be targeted by multiple other RNAs. In the last years the miRNA mediated mRNA expression was investigated and shown to be a key biological mechanism that is involved in gene expression, as miRNAs tend to bind o the 3′ untranslated region (3'UTR) of mRNAs and influence gene expression.^19^, ^20^ Thus, the interaction between non‐coding RNAs and *F8* gene might represent an important path in understanding how patients without *F8* mutations are lacking FVIII in blood and develop HA. It was shown that miR‐374b‐5p and miR‐30c‐5b target *F8* gene and impair FVIII, indicating that classical HA could be evaluate not only by the mutational status.^5^, ^21^

## 4 DISCUSSION

In the case of HA, miRNAs shown direct involvement in the down‐regulation of *F8* gene and many studies were indicating that other genes that encode coagulation factors can be modulated by miRNAs thus triggering bleeding disorders and thrombosis.^19^, ^22^

AH is characterized by the autoantibodies that neutralize FVIII by inhibiting *F8* and occurs in patients without bleeding issues in family and the disease is developed during lifetime, most of the patients being old and frail, with other comorbidities. Two cases of AH were described as *F8* mutant with point mutation. Thus, an epigenetic evaluation of AH cases could bring new diagnostic/detection methods in the spotlight.^10^, ^23^ AH may be triggered by other autoimmune diseases, drug side effects, different types of cancer; however, some of AH cases remain idiopathic and deep investigations are needed to improve patient management to avoid ICU hospitalisation due to haemorrhage situations.^24^, ^25^, ^26^ The evaluation of the transcriptome could indicate which transcripts can be associated with AH and if they can represent a potential biomarker for it.

Samples from two patients with type A haemophilia were sequenced and the expression of miRNAs was evaluated. For both analysed samples no *F8* gene mutations were reported. However, the results indicate that two miRNAs, miR‐19b‐3p and miR‐186‐5p could target the 3'UTR of *F8* gene and modulate the FVIII protein level. The involvement of miR‐19b‐3p and miR‐186‐5p in haemophilia was previously reported by Jankowska et al., among several other miRNAs detected in patients' samples.^21^ As presented in Figure 3, both miRNAs had higher expression in the acquired haemophilia samples compared to healthy and mild haemophilia, while in the comparison with other groups the expression was less intense. Moreover, the interaction of miRNAs and 3'UTR of *F8* gene was evaluated in murine models, the study conducted by Jankowska reported miR‐208a, miR‐351 and miR‐125a as modulators of *F8* gene by their interaction with 3'UTR of *F8* gene,^27^ furthermore similar study reported 52 miRNAs that interact with *F7*, *F8* and other haemostatic‐associated genes.^28^

The herein presented results open a new perspective in the detection of new biomarkers for acquired haemophilia, by making comparisons between AH and healthy donor samples and classical haemophilia. We used plasma collected from healthy donors and patients suffering both of AH and classical haemophilia and small RNA fragments were extracted for RNA sequencing, thus a screening on small non‐coding RNAs was made for each sample. As presented in Figure 1, the plotting was not significant on PCA due to the low number of samples which represent our main study limitation, however by using the bioinformatic tools we were able to evaluate the most relevant small RNAs identified in each sample. As represented in Figure 2, comparisons were made between all experimental groups and the heatmap highlighted the most significant small RNAs that were increased or decreased in each sample. With a FC cutoff of two, several molecules with different expression were observed mostly at comparisons between AH and all other groups. Thereafter, we displayed in a Venn Diagram the only molecule that was common and upregulated in AH compared to all other groups, haemoglobin subunit alpha 1 (HBA1‐201), indicating the probability of a compensatory physiological mechanism to blood loss.

## 5 CONCLUSION

While classical haemophilia is X‐linked disease and occurs due to mutations in *F8* gene, acquired haemophilia is developed due to biological mechanisms that can be epigenetically modulated by different factors such as non‐coding small RNAs. Both cases of acquired haemophilia A were idiopathic, with not underlying medical condition. Even if the demographic characteristics of both patients were similar, we completely understand that two cases are not truly relevant to draw any definitive conclusion, but acquired haemophilia A is an ultra‐rare disease, especially idiopathic cases, that did not have any pre‐existent malignancy, any endocrinologic, infectious or surgical history. Still, investigations regarding this aspect are needed to discover new biomarkers for acquired haemophilia.

## AUTHOR CONTRIBUTIONS

**Adrian‐Bogdan Tigu:** Data curation (equal). **Ionut Hotea:** Data curation (equal). **Rares Drula:** Investigation (equal). **Alina‐Andreea Zimta:** Investigation (equal). **Noemi Dirzu:** Methodology (equal). **Maria Santa:** Investigation (equal). **Delia Dima:** Investigation (equal). **Catalin Constantinescu:** Investigation (equal). **Jon‐Thor Bergthorsson:** Investigation (equal). **Victor Greiff:** Investigation (equal). **Diana Gulei:** Investigation (equal). **Daniel Coriu:** Investigation (equal). **Margit Serban:** Investigation (equal). **Johnny Mahlangu:** Methodology (equal). **Ciprian Tomuleasa:** Supervision (equal); visualization (equal).

## FUNDING INFORMATION

IH is funded by an internal grant of the Iuliu Hatieganu University – School of Doctoral Studies. BT is supported by a national grant of the Romanian Academy of Scientists (Academia Oamenilor de Stiinta din Romania) 2023–2024. ABT, DG, JTB and VG are supported by an international collaborative grant of the European Economic Space between Romania and Iceland 2021–2023: ‘Cooperation strategy for knowledge transfer, internationalization and curricula innovation in the field of research education at the 3rd level of study –AURORA.’. The experiments were funded by an international grant awarded by the Novo Nordisk Haemophilia Foundation to the Romanian Haematology Society—Romania 4. CT is supported by a grant by grants awarded by the Romanian National Ministry of 350 Research, Innovation, and Digitalisation: Project PN‐III‐P4‐ID‐PCE‐2020‐1118.

## CONFLICT OF INTEREST STATEMENT

The authors declare no conflicts of interest.

## Data Availability

All data in available upon request.
